# Women Representation and Gender Equality in Different Academic Levels in Veterinary Science

**DOI:** 10.3390/vetsci8080159

**Published:** 2021-08-07

**Authors:** Xinyue Liu, Rebecca Dunlop, Rachel Allavena, Chiara Palmieri

**Affiliations:** School of Veterinary Science, The University of Queensland, Gatton, QLD 4343, Australia; xinyue.liu1@uq.net.au (X.L.); r.dunlop@uq.edu.au (R.D.); r.allavena@uq.edu.au (R.A.)

**Keywords:** gender equality, academia, veterinary science

## Abstract

Women’s participation and completion at veterinary schools has increased globally for the past few decades. However, increased female graduates have not translated into similar patterns of academic staffing. The gender distribution within each academic level at eight accredited veterinary faculties in Australia and New Zealand, 38 accredited faculties in the USA and Canada and 98 accredited faculties in Europe were analyzed. Women occupied 47.9%, 45.5% and 47.5% of the academic positions in Australia/New Zealand, the USA/Canada and Europe, respectively. Compared to their male counterparts, female academics were more likely to hold the lower ranked positions. The gender distribution is skewed toward men in the senior positions at or above associate professor level in all analyzed regions. The findings of this study confirm gender inequality in academic progression meaning there is a continued need to develop strategies to eliminate inequity in veterinary science faculties worldwide.

## 1. Introduction

The existence of gender bias and inequity in academia has been highlighted as a significant issue, meaning effective measures and interventions should be applied to mitigate this societal problem [1]. We are all familiar with the famous “scissor graph” which depicts the reality of gender imbalance in senior roles, especially in STEM (science, technology, engineering and mathematics) disciplines [2]. Gender disparities in academia have been further amplified by the COVID-19 pandemic with the proportion of research published by women gradually decreasing and overall lower research productivity of female academics compared to men [3,4,5]. In Australia, gender equality in academia has been monitored since the mid-1980s [6]. To remove sex discrimination, and increase women’s representation in academia, Australian universities have made dramatic changes, prompted by government legislation, regulatory frameworks, university gender equity strategies and other strategic measures. By 2014, women represented 44% of the academic staff in Australian universities, holding 31% of the senior positions [6]. Gender equality tends to occur in junior to middle level academic positions in Australia, whereas women are still under-represented in the more senior positions in most of the prestigious institutes, particularly in the field of natural sciences and engineering [7]. In New Zealand, the 2012 New Zealand Census of Women’s Participation (NZCWP)—an initiative of the Human Rights Commission and the New Zealand Centre for Women and Leadership in place since 2004—highlighted that only 18.7% of the professors in the eight universities were female [8]. As a key takeaway from the NZCWP website, “women’s low representation at the top, despite increasing participation at entry levels, remained systemic and frustrating after 10 years of tracking the data”.

The under-representation of women in academia is also a major issue in the USA and Canada, where female academic staff are found to occupy lower ranked academic positions compared to male faculty. The differences in the senior positions are particularly significant. According to the American Association of University Professors [9], males outnumbered female academics by 41% at the full professor level [10,11]. Women further have relatively minor representation in STEM disciplines [10].

In the EU, there is also a significant gap between female and male representation in senior academic positions, particularly in the STEM disciplines. When examined across individual nations in the EU, the percentage of female academic staff in full professor positions ranged from 13% to 54.3% according to the European Commission [12]. The female academics in the senior positions were found to be concentrated in humanities, social and medical sciences, but occupied relatively few positions in engineering, technology and natural sciences [12]. Similarly, the female academics in the UK also tended to be in lower ranked positions. The data were in line with the “the higher the fewer” pattern in the academic career progression of women, a phrase used to describe the decreased proportion of women at every rung of the academic ladder [10,13]. 

The gender inequality in academia can be explained by a confluence of family circumstances, promotion systems and professional networks. Firstly, women are constrained in the academic career development due to the fact that they have to contend with parenting and housework [14]. Women are two times more likely to leave an academic career compared to men [15,16] or change academic positions [17], and less likely to be awarded tenure than men [18]. Secondly, there is gender bias in the allocation of work to female academics, who are likely to be assigned proportionately more teaching and administrative tasks, and less research opportunities than their male counterparts. As research productivity is given greater emphasis than teaching activities in the promotion in academia, this negatively impacts female career progression [8,19,20]. Further, the criteria for promotion, such as numbers of publications, success in grant applications and student evaluations are prone to gender bias [1]. Women academics in STEM have lower publication rates than men [21], most likely explained by the different career lengths with higher dropout rates, more career interruptions and shorter publishing career compared to men [22]. Women are also disadvantaged in grant peer reviews, with male investigators viewed more easily as scientific leaders than women and their applications scored more competitively and positively than applications led by female investigators [23,24]. In Australia, for example, a review of the 2019 Australian Research Council (ARC) data has highlighted that only 27.5% of women applied to the ARC funding schemes overall and 2307 males secured funding in comparison to 939 women [25]. Similarly, promotion panels tend to privilege male disciplinary work over female, resulting in a gap between the promotion rate of men and women [6,26]. Lastly, male academics tend to take advantage of male networks to obtain more favorable workloads and achieve more collaboration, thus lowering the visibility of women academics [20].

Studies have been conducted to reveal the gender bias and inequity in academic positions in different disciplines, in particular in the medical and biological science field, and the research and knowledge on gender diversity in academia is steadily increasing. However, data on veterinary science is limited. According to previous studies, although an increased number of women study and work in the field of veterinary science, the culture still remains stereotypically masculine [27,28].

This study therefore aimed to evaluate, and compare, the proportion of female academic staff in veterinary science faculties in Australia and New Zealand, Europe and North America. The study assesses workplace gender distribution within each academic level and compares distributions between academic levels in different veterinary science faculties and countries. Thus, this work contributes detailed global analysis on gender distribution academia in the field of veterinary science. 

## 2. Materials and Methods

### 2.1. Data Collection

The list of veterinary science faculties in Australia, New Zealand, the USA and Canada was obtained from an established online veterinary educational resource platform (https://en.wikivet.net/Vet_Schools_Worldwide; accessed on 28 November 2020). Faculties without an available list of academic staff were excluded from this study. In total, data from eight accredited veterinary faculties in Australia and New Zealand, 38 accredited faculties in the USA and Canada and 98 accredited faculties in Europe were included in the study. The Australia/New Zealand faculties were accredited by the Australasian Veterinary Boards Council Inc. (AVBC), and the USA/Canada faculties were accredited by the American Veterinary Medical Association (AVMA). Regarding the European faculties, 85 of them were accredited by the European Association of Establishments for Veterinary Education (EAEVE), while the remaining 13 faculties were not accredited. In order to obtain more accurate results, all these faculties were taken into account and grouped according to the geographical subregions of Europe (Table 1). 

The collected information included full name, gender and academic position of the academic staff engaged in either teaching or research duties. All searches and data collection were performed in 30 December 2020. The data were then entered into Excel spreadsheets for further data coding and consolidation.

### 2.2. Gender Inference

The gender of the individuals was inferred by checking the photograph or gender-specific pronoun (such as “he” or “she”) on the official websites of the veterinary science faculty. In this study, it is relatively limiting that the binary classification of gender was adopted despite the existence of other gender identities such as transgender, intersex, agender and many others. 

Since the situation existed that no gender information was available on the website of some faculties, a tool called genderize.io (https://genderize.io, accessed on 30 December 2020) was used to predict their gender. If the probability was less than 0.95, the online databases and personal web pages (such as Google Scholar, ResearchGate, LinkedIn, Facebook and Twitter) were used to help with the inference of binary gender.

### 2.3. Data Coding and Consolidation

For the veterinary science faculties of Australia/New Zealand and the USA/Canada regions, the academic titles were coded using the abbreviations as shown in Table 2 and Table 3. The numbers of females and males in each academic position were calculated and recorded.

The academic titles in Europe varied from country to country. It was also inaccurate to directly translate the academic titles from the original languages to English, since the same translated academic title did not necessarily refer to the same academic position or level in different countries. Due to the complexity of using the equivalence between the international academic positions, the academic titles were first reclassified into three categories, including junior, intermediate and senior levels, based on the classification system of each country according to the European Eurydice Report [29]. The data within the same category was then ready for analysis and comparison.

### 2.4. Data Analysis

Each academic was either allocated a 1 (male) or 0 (female). For all analyses, general linear models were used, assuming a binomial distribution and with gender as the binomial response variable. The null model assumed the probability of any academic to be male a value of 0.5 (i.e., an even distribution of males and females within each academic level). The model was tested for significance outside this in terms of gender distribution.

Here, we tested the hypothesis that, as academic level increased, gender distribution was increasingly more likely to be skewed towards males. Academic positions within each region were recategorized into an ordered variable (termed academic level) according to the definition of each academic position. Separate models were generated for each region, as academic positions and levels were defined differently within each region. As the predictor variable was ordered, model coefficients specified the effect of this variable as linear, quadratic, cubic and, if applicable, higher-order contrasts. Model results were plotted as expected probabilities using the modelled relationship (linear, quadratic or polynomial) in the accompanying figures. 

## 3. Results

### 3.1. Australia/New Zealand

In total, there were 603 data points from the Australia/New Zealand region (Table 4) comprising of 289 females and 314 males. Female academics constituted 47.9%, approximately half of the faculty. However, females only occupied 38.0% of the associate professor and professorial positions within this region (Table 4).

**Figure 1:**
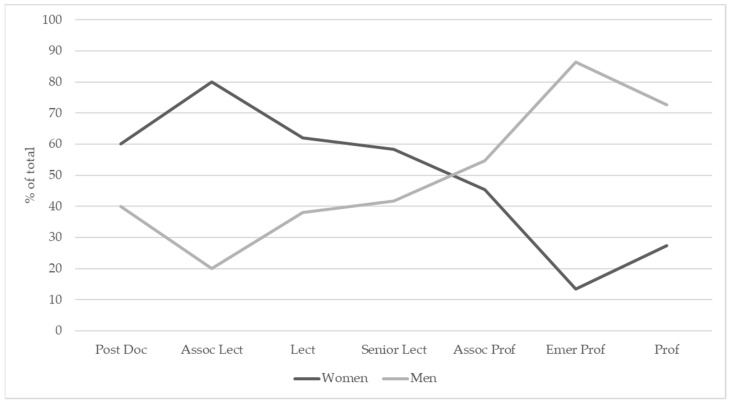
Scissor diagram showing the gender distribution within different academic levels in Australian and New Zealand veterinary schools.

In general, the gender distribution was skewed towards females at associate professor, senior lecturer, lecturer, postdoc. research fellow, associate lecturer and Tutor levels, and towards males at professor and emeritus professor levels (Figure 1). 

**Figure 2:**
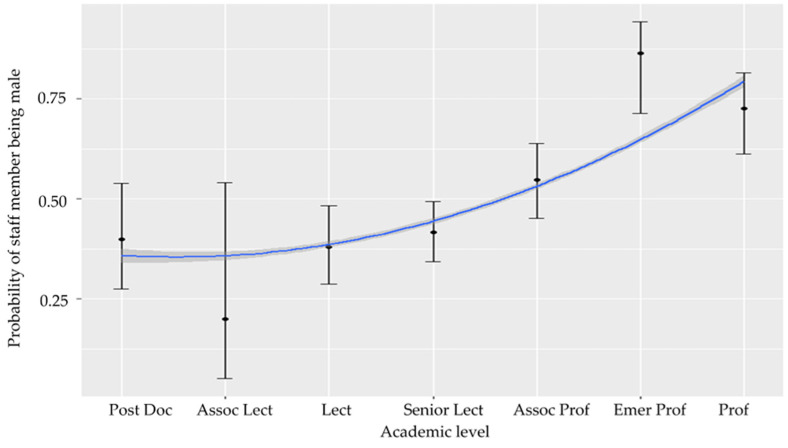
Modelled probabilities of a staff member being male as a function of academic level in Australia/New Zealand. The modelled quadratic term (blue line) is also plotted which includes the standard error (dark grey).

For the analysis, Tutor and adjunct positions were eliminated due to the small sample size and uncertainty of position meaning 531 data points remained. Model results suggested no significant linear relationship between the gender distribution within each academic level. There was, however, a significant relationship if modelled as a quadratic term (z = −2.473, *p* = 0.013). This is because, Postdoc., lecturer and senior lecturer level (levels 1, 3 and 4), were dominated by females (with between 30 and 40% male), and with very little difference in the gender distribution of staff between these levels (Figure 2). At associate lecturer (level 2), male representation was only 25%. Male representation at academic levels of 5 (associate professor) and above increased to over 50%, peaking at 85% within the emeritus professor position (Figure 2). Almost 75% of professors (level 7) were male. In other words, there was a significant increase in the likelihood of a staff member being male in levels 5, 6 and 7 (Figure 2). Academic positions classified as higher levels were more likely to contain over 50% male in the Australian/New Zealand region.

### 3.2. USA/Canada

**Figure 3:**
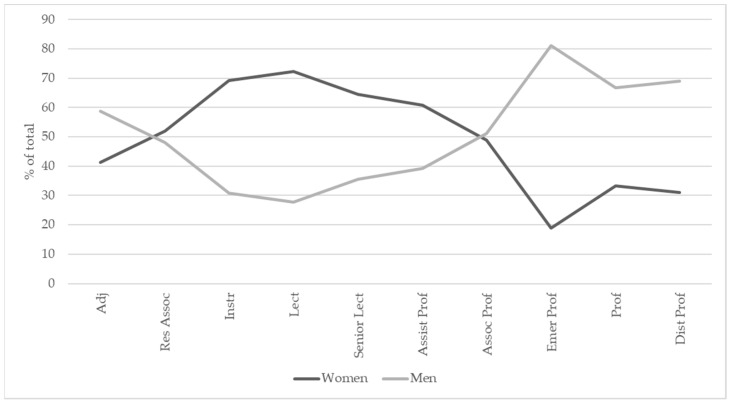
“Double” scissor diagram showing the gender distribution within different academic levels in USA/Canada veterinary schools.

There were 5162 data points in the USA/Canada. However, the sample size of the visiting faculty was too small (*n* = 8), so this group was eliminated from data analysis. A total of 5154 observations remained comprising of 2334 females (45.3%) and 2820 males (54.7%). Females held 39.9% of the positions above associate professor (excluded emeritus position) (Table 5, Figure 3).

**Figure 4:**
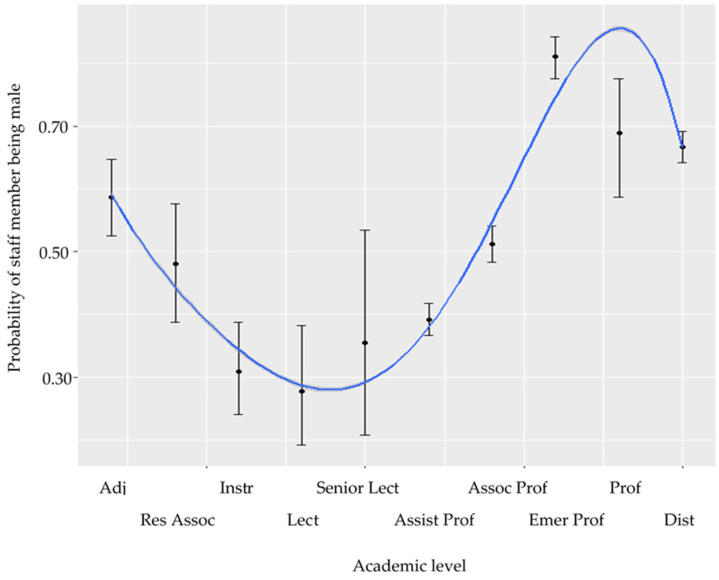
Modelled probabilities of a staff member being male as a function of academic level in the USA/Canada region. The modelled polynomial term (blue line) is also plotted which includes the standard error (dark grey).

For this region, academic positions were recategorized into the ordered variable, where 1 = adjunct faculty, 2 = research associate, 3 = instructor, 4 = lecturer, 5 = senior lecturer, 6 = assistant professor, 7 = associate professor, 8 = emeritus professor, 9 = professor and 10 = distinguished professor. This time the linear relationship between gender distribution and academic level was significant in the USA/Canada region (z = −2.82, *p* = 0.005), indicating a linear increase in the number of male staff members with increasing academic level. However, the relationship was more complex, and better explained by a polynomial term (z = −6.640, *p* < 0.0001). There was a significant reduction in male representation between level 1 (adjunct faculty with 60% of staff being male), and levels 2, 3 and 4 (research associate, instructor and lecturer) where only 30% of instructors and lecturers were male. Male representation then increased to become an even gender distribution (50% male/female) by level 7 (assistant professor). Levels 8, 9 and 10 (emeritus professor, professor and distinguished professor) were dominated by males, with the professor category being approximately 80% male (Figure 4).

### 3.3. Europe

**Figure 5:**
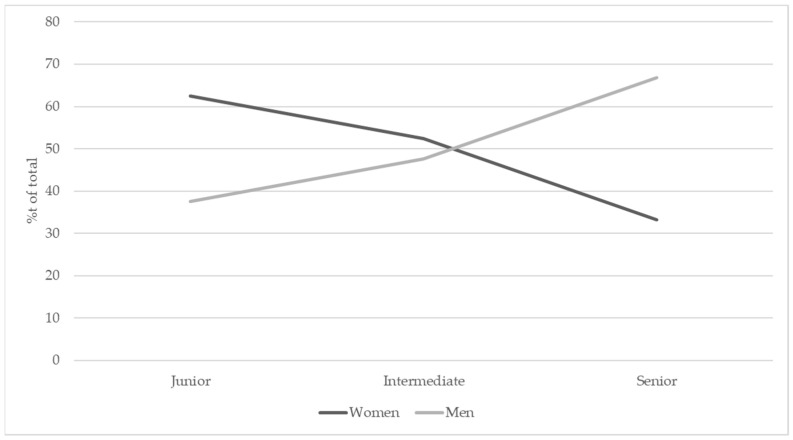
Scissor diagram showing the gender distribution within different academic levels in European veterinary schools.

In total, there were 9098 observations for the Europe region, comprising of 4336 females (47.6%) and 4762 males (52.3%) (Table 6, Figure 5). Data were grouped by geographical subregion as shown in Table 7 given academic positions were comparable between European regions. Usually, the senior academic staff are professors, intermediate staff are associate professors and assistant professors, and the junior staff are lecturers, research fellows and assistants. In some countries, associate professor is also classified into the senior category. In general, 47.7% of the academic positions were held by females, but in the senior level, there was approximately twice the number of males compared to females.

**Figure 6:**
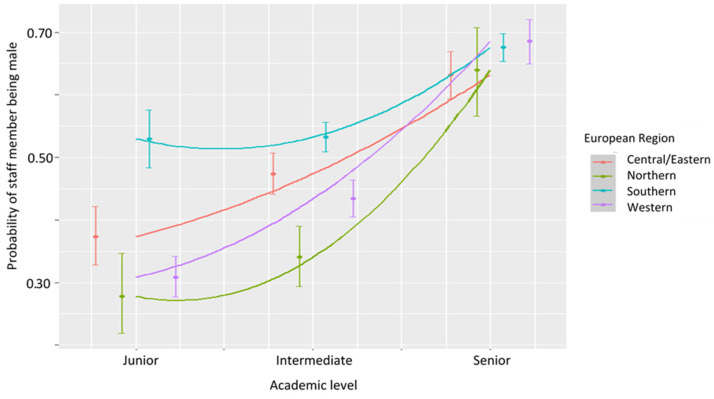
Modelled probabilities of a staff member being male as a function of academic level in the European regions. The modelled quadratic term (blue line) is also plotted for each region which includes the standard error (dark grey).

For this analysis, the European subregion was included as an extra term to determine if there were trend differences between European regions. The overall trend was, as with other regions, male representation significantly increased with an increase in academic level (z = 6.021, *p* < 0.0001). However, there were significant differences in this relationship with region. Northern (z = 2.795, *p* = 0.005), Western (z = 5.463, *p* < 0.0001) and Central and Eastern Europe (z = 2.778, *p* = 0.005) showed the significantly different trend to Southern Europe. This because academic staff within these three regions were highly female-dominated at junior level and highly male-dominated at the senior level. In comparison, academic staff based in Southern Europe had an almost 50% distribution between males and females at the junior level, with a similar distribution of males and females to other European regions at senior level. In other words, there was a more profound shift in Northern, Central/Eastern and Western Europe to a male-dominated distribution compared to Southern Europe (Figure 6). 

## 4. Discussion

In the past few decades, veterinary science has experienced feminization, as the number of women studying at veterinary schools increased dramatically [27]. Nevertheless, the relatively high levels of female participation and completion of veterinary degrees have not translated into a similar gender trend in academic staffing, potentially due to the gendered masculine culture and multiple barriers to women progressing in academia [30].

In Australia/New Zealand, the USA/Canada and Europe, female academics accounted for almost half of the veterinary science faculty members, being between 45.5% and 47.9%. However, the gender distribution was not even within the academic levels. In general, female academics were overrepresented in junior levels and underrepresented at senior ranks. For the faculties in Australia/New Zealand, results suggested that females significantly outnumbered their male counterparts in the low positions but were significantly under-represented as the level increased to associate professor and above. In the USA/Canada, the relationship between academic level and gender distribution was also significant, but more complex than in Australia/New Zealand. For the USA/Canada academics, the gender distribution was nearly even in the entry level, then dominated by females in junior and intermediate levels, returning to equal and finally strongly skewed towards males by professor and distinguished professor levels. As for Europe, the association between academic level and gender was significantly different within the geographical subregions. The shift from female-dominated to male-dominated distribution as the academic level increased was more profound in Northern, Western and Central and Eastern Europe than in Southern Europe. This is because, in Southern Europe, gender distribution at entry level was almost even, but female-dominated in the other European regions. In summary, the current study revealed that the gender equality in academic position, particularly the senior positions, has not been achieved in veterinary science within the three major global geographic regions investigated.

These results are consistent with the previous data of a total academic workforce of 383,424 academics from the USA reported by Diezmann and Grieshaber [10]. Despite females dominating the workforce at instructor and lecturer level, female representation declined level by level to the professor level indicating a significant lack of gender equity in the analyzed faculties [10]. Another study on gender distribution with academic rank among faculty surgeons at veterinary schools in the USA found that female academics were concentrated in lower academic positions, and male academics were 2.5 times more likely to be associate professor or professor than their female counterparts [30]. The current study shows the same trend in Australia/New Zealand and Europe with notable similarity in gender distribution across all analyzed regions. At lower academic positions, across all regions, females comprised about 70% of the workforce. At more senior positions, females comprised about 30% of the workforce. As is mentioned by the European Commission [12], various measures have been adopted to improve the representation of women in senior positions, such as leadership training, gender equality plans and human resources strategy for researchers. However, the changes are not significant, and much work remains to eliminate the gender inequity in academia [10,12]. 

There are few limitations of this study. Firstly, the academic staff list of some veterinary science faculties in Europe was unavailable or incomplete on the official web pages due to privacy considerations. However, despite some omissions we were able to obtain a large sample size improving the accuracy of the results. Secondly, the academic rank of individual academics changes over time where individuals generally increase in rank over their career span. Therefore, there was a risk that the data source pages did not provide the most up-to-date information. Thirdly, this study adopted the binary classification of gender, as part of the study methodology, but the authors recognize there are non-binary gender identities such as transgender, intersex and agender. Future work could explore self-identification of gender for a more accurate picture. 

Having confirmed a broad geographic gender gap across academic rank in veterinary science, there are several issues for future consideration. Future studies should explore factors leading to the disproportionately low levels of women academics in senior levels in veterinary academia, and what measures and strategies should be taken to reduce the bias and inequity. Interventions that should be implemented to reduce inequities for women in veterinary science faculties and overall in the STEM academic fields should be focused on (a) changing and expanding the recruitment efforts to reach a diverse applicant pool, (b) provide mentoring and professional development opportunities in order to retain and promote women and prepare them to be the future faculty leaders and (c) creating strategic communication focused on equality goals.

There are many specialty areas in veterinary science, and the gender distribution in each field is likely to be different, warranting further investigation. Having confirmed consistent global gender imbalance in veterinary academia, further study into the factors producing gender variance in academic rank is warranted in order to define pathways to correct gender imbalance. 

## Figures and Tables

**Table 1:** Geographical subregions of Europe.

Central and Eastern Europe	Northern Europe	Southern Europe	Western Europe
Bosnia and Herzegovina	Denmark	Greece	Austria
Bulgaria	Estonia	Italy	Belgium
Czech Republic	Finland	Portugal	France
Croatia	Latvia	Spain	Germany
Hungary	Lithuania	Turkey	Ireland
Poland	Norway		Netherlands
Romania	Sweden		Switzerland
Serbia			United Kingdom
Slovakia			
Slovenia			

This table only includes the countries of the veterinary science faculties included in the study.

**Table 2:** Abbreviation codes for academic position in Australia/New Zealand.

Academic Position	Abbreviation Code
Professor	Prof
Associate Professor	Assoc Prof
Senior Lecturer	Senior Lect
Lecturer	Lect
Postdoc. Research Fellow	Post Doc
Associate Lecturer	Assoc Lect
Emeritus Professor	Emer Prof
Adjunct faculty	Adj
Tutor	Tutor

**Table 3:** Abbreviation codes for academic position in the USA/Canada.

Academic Position	Abbreviation Code	Inclusion
Distinguish	Dist Prof	Distinguished Professor, Endowed Professor
Professor	Prof	Professor, Clinical Professor, Research Professor, Teaching Professor
Associate Professor	Assoc Prof	Associate Professor, Associate Teaching Professor, Associate Research Professor
Assistant Professor	Assist Prof	Assistant Professor, Assistant Teaching Professor, Assistant Research Professor, Assistant Clinical Professor
Senior Lecturer	Senior Lect	Senior Lecturer
Lecturer	Lect	Lecturer, Clinical Lecturer
Instructor	Instr	Instructor, Clinical Instructor
Research Associate	Res Assoc	Research Associate, Postdoctoral Research Associate
Emeritus Professor	Emer Prof	Emeritus Faculty
Adjunct faculty	Adj	Adjunct Faculty, Joint Professor, Affiliate Professor
Visiting faculty	Vis	

**Table 4:** Gender distribution across different academic levels in Australia/New Zealand.

Academic Level Data	Female		Male	
	#	%	#	%
Prof	20	27.4	53	72.6
Assoc Prof	48	45.3	58	54.7
Senior Lect	95	58.3	68	41.7
Lect	57	62.0	35	38.0
Post Doc	30	60.0	20	40.0
Assoc Lect	8	80.0	2	20.0
Emer Prof	5	13.5	32	86.5
Adj	23	33.8	45	66.2
Tutor	3	75.0	1	25.0

**Table 5:** Gender distribution across different academic levels in the USA/Canada.

Academic Level Data	Female		Male	
	#	%	#	%
Dist Prof	28	31.1	62	68.9
Prof	462	33.3	924	66.7
Assoc Prof	542	48.8	569	51.2
Assist Prof	859	60.8	554	39.2
Senior Lect	20	64.5	11	35.5
Lect	60	72.3	23	27.7
Instr	105	69.1	47	30.9
Res Assoc	55	51.9	51	48.1
Emer Prof	101	18.9	434	81.1
Adj	102	41.3	145	58.7
Vis	4	50	4	50

**Table 6:** General gender distribution across different academic levels in the Europe region.

Academic Level Data	Female		Male	
	#	%	#	%
Senior	1069	33.2	2147	66.8
Intermediate	2130	52.4	1933	47.6
Junior	1137	62.5	682	37.5

**Table 7:** Gender distribution across different academic levels in European subregions.

Academic Level	Northern				Western				Southern				Central and Eastern			
	F		M		F		M		F		M		F		M	
	#	%	#	%	#	%	#	%	#	%	#	%	#	%	#	%
Senior	63	36	112	64	209	31.4	456	68.6	573	32.4	1194	67.6	224	36.8	385	63.2
Intermediate	246	66	127	34	607	56.6	465	43.4	802	46.8	913	53.2	475	52.6	428	47.4
Junior	135	72.2	52	27.8	533	69.1	238	30.9	212	47	239	53	257	62.7	153	37.3

F = female; M = male.

## Data Availability

The data presented in this study are available on request from the corresponding author.
